# Al Doping Effect on Enhancement of Nonlinear Optical Absorption in Amorphous Bi_2_Te_3_ Thin Films

**DOI:** 10.3390/ma18061372

**Published:** 2025-03-20

**Authors:** Tengfei Zhang, Shenjin Wei, Shubo Zhang, Menghan Li, Jiawei Wang, Jingze Liu, Junhua Wang, Ertao Hu, Jing Li

**Affiliations:** 1 Department of Optical Science and Engineering, Shanghai Ultra-Precision Optical Manufacturing Engineering Center, Fudan University, Shanghai 200433, China; 21110720020@m.fudan.edu.cn (T.Z.); shenjin_wei@fudan.edu.cn (S.W.); 20110720017@fudan.edu.cn (S.Z.); 23110720009@m.fudan.edu.cn (M.L.); 24110720161@m.fudan.edu.cn (J.W.); 24210720001@m.fudan.edu.cn (J.L.); 2 Shanghai Frontiers Science Research Base of Intelligent Optoelectronics and Perception, Institute of Optoelectronics, Fudan University, Shanghai 200438, China; wangjunhua@fudan.edu.cn; 3 College of Electronic and Optical Engineering and Jiangsu Province Engineering Research Center for Fabrication and Application of Special Optical Fiber Materials and Devices, Nanjing University of Posts and Telecommunications, Nanjing 210023, China; iamethu@njupt.edu.cn

**Keywords:** Al-doped Bi_2_Te_3_ thin films, magnetron co-sputtering, open-aperture Z-scan, nonlinear optical absorption, damage threshold

## Abstract

Bismuth telluride (Bi_2_Te_3_) has attracted significant attention due to its broadband ultrafast optical response and strong nonlinearity at high laser fluence in the field of optoelectronic materials. The objective of this work is to study the effect of Al doping on the structure, linear optical properties, and nonlinear optical absorption behavior of Bi_2_Te_3_ thin films. The amorphous Al-doped Bi_2_Te_3_ thin films with varying Al doping concentrations were prepared using magnetron co-sputtering. The structure and linear optical properties were characterized using X-ray diffraction, scanning electron microscopy, energy-dispersive X-ray spectroscopy, X-ray photoelectron spectroscopy, spectroscopic ellipsometry, and UV/Vis/NIR spectrophotometry. The third-order nonlinear optical absorption properties of Al: Bi_2_Te_3_ thin films were investigated using the open-aperture Z-scan system with a 100 fs laser pulse width at a wavelength of 800 nm and a repetition rate of 1 kHz. The results indicate that Al dopant reduces both the refractive index and extinction coefficient and induces a redshift in the optical bandgap. The optical properties of the films can be effectively modulated by varying the Al doping concentration. Compared with undoped Bi_2_Te_3_ thin films, Al-doped Bi_2_Te_3_ thin films exhibit larger nonlinear optical absorption coefficients and higher damage thresholds and maintaining high transmittance. These findings provide experimental evidence and a reliable approach for the further optimization and design of ultrafast nonlinear optical devices.

## 1. Introduction

Bi_2_Te_3_ is a typical V_2_VI_3_ binary chalcogenide semiconductor with a layered crystal structure belonging to the rhombohedral system [1]. It consists of a layered system with five atomic layers as the basic unit, referred to as the quintuple layer [2]. The atomic sequence within these quintuple layers is arranged as Te(1)-Bi-Te(2)-Bi-Te(1), where the numbers in parentheses distinguish the inequivalent Te sites. The Te-Bi bonds within the quintuple layer are polar covalent, while only weak van der Waals interactions exist between Te(1)-Te(1) planes for neighboring quintuple layers [3]. This unique layered structure not only endows Bi_2_Te_3_ materials with unique photoelectric properties [4] but also makes them exhibit excellent thermoelectric properties [5]. Consequently, Bi_2_Te_3_ has demonstrated broad application prospects in thermoelectric materials, optoelectronic devices, and ultrafast nonlinear optics.

For applications, Chowdhury et al. integrated thermoelectric coolers fabricated from nanostructured Bi_2_Te_3_-based superlattice films into advanced electronic packages, achieving localized cooling of up to 15 °C on a silicon chip under a high heat flux of approximately 1300 W cm^−2^ [6]. This technology enables dynamic and site-specific cooling by integrating multiple thermoelectric devices that can be activated as needed, with potential applications in various fields such as DNA microarrays and hybrid vehicle battery cooling. Qiao et al. proposed a graphene-Bi_2_Te_3_ heterostructure photodetector that significantly enhanced photoresponsivity and sensitivity. The detection wavelength range was further extended to the near-infrared and telecommunication bands, overcoming the limitations of pure graphene-based devices [7]. Xiao et al. confirmed room-temperature ferromagnetism in undoped nanostructured Bi_2_Te_3_ topological insulators and, based on first-principles calculations, demonstrated that the intrinsic point defect of the anti-site Te is the cause of the magnetic moment, providing new insights into spintronics and quantum computing [8]. In the field of nonlinear optics, Jung et al. demonstrated a femtosecond mode-locked all-fiber laser operating in the 2 μm region using a bulk Bi_2_Te_3_ topological insulator as a saturable absorber, achieving a stable ultrafast pulse with a temporal width of approximately 795 fs [9]. Furthermore, Chen et al. utilized the high modulation depth of Bi_2_Te_3_ to generate stable Q-switched pulses with per-pulse energy up to 1.5 μJ in a passively doped erbium fiber laser. Its broadband saturable absorption characteristics were conducive to tunable operation from 1510.9 nm to 1589.1 nm [10].

It is evident that Bi_2_Te_3_ holds significant potential for application across multiple disciplines, including thermoelectric, topological, infrared, and nonlinear optical research. As a result, increasing attention has been directed in recent years towards methods for further optimizing and tuning the performance of Bi_2_Te_3_. Among these, elemental doping as one of the most effective and commonly used approaches, has been extensively explored. For instance, Ren et al. (2018) enhanced the stability of amorphous Bi_2_Te_3_ for phase-change memory applications through nitrogen doping, and observed smaller defective grains and abnormal volume expansion after crystallization [11]. Han et al. explored the effect of Pb-CuI co-doping on the thermoelectric performance of Bi_2_Te_3_ and found that Pb significantly reduced the carrier concentration and increased the Seebeck coefficient, while 1% CuI-Pb co-doped Bi_2_Te_3_ achieved the best thermoelectric properties with a figure of merit (ZT) of 0.96 at 370 K [12]. In further studies on its thermoelectric properties, Ga-doped Bi_2_Te_3_ demonstrated an enhancement in power factor. Specifically, doping with 2 mmol of Ga increased the ZT value to 1.1 at 50 °C, while higher doping concentrations led to a decline in performance [13]. The electronic structures of La- or Ce-doped Bi_2_Te_3_ were analyzed using density functional theory and the Boltzmann transport equation. La doping increased the Seebeck coefficient of Bi_2_Te_3_, while Ce doping exhibited a similar effect, providing insights into the transport properties of La- or Ce-doped Bi_2_Te_3_-based thermoelectric materials [14]. In addition, Mn doping reduced the carrier density and converted the carrier type of Bi_2_Te_3_ films to p-type conduction. The structure and optical constants of the film were significantly influenced, resulting in higher transmittance and lower reflectance [15]. Previous studies have extensively investigated the impact of metallic element doping on the nonlinear optical properties of other chalcogenide compounds, such as MoS_2_ [16], Sb_2_Se_3_ [17], and InTe [18], yielding remarkable results. These studies demonstrate that appropriate dopants can introduce additional electronic states and modify the band structure, thereby optimizing the nonlinear optical properties of the semiconductor materials. However, the effects of metallic element doping on the nonlinear optical absorption and laser damage properties of Bi_2_Te_3_ thin films remain inadequately explored. A deeper understanding of the modulation mechanisms of elemental doping on the nonlinear optical properties is crucial for the development of high-performance nonlinear optical materials. This advancement holds significant scientific implications for pioneering next-generation high-power lasers, ultrafast optical switching devices, and precision laser processing systems.

For nonlinear optical devices, semiconductor materials with excellent optical transmittance, strong nonlinear optical responses, and high damage thresholds are critical in various applications. However, the simultaneous optimization of these three key properties often presents significant challenges. As demonstrated by Jin et al., the saturable absorption properties of MoS_2_ films were enhanced by increasing the thickness, which was accompanied by a reduction in transmittance and led to additional energy loss [19]. In our previous studies, the crystallinity and third-order nonlinear optical absorption coefficient of Bi_2_Te_3_ thin films were improved by vacuum annealing treatment, but this process was accompanied by an increase in its linear absorption coefficient, causing a decrease in its transmittance and the damage threshold [20]. To address these challenges, this study focuses on the regulation of nonlinear optical properties in Al-doped amorphous Bi_2_Te_3_ thin films, aiming to achieve simultaneous enhancement of third-order nonlinear optical absorption coefficients, damage thresholds, and transmittance. Al is a typical light metal present in a +3-valence state (Al^3+^) throughout the sample, which exactly matches the valence state of A_2_B_3_-type chalcogenides. Therefore, Al-doped Bi_2_Te_3_ thin films can achieve micro-structure regulation without changing their configuration, thereby adjusting their macroscopic physical properties. Based on the comprehensive characterization of structural, chemical, and optical properties in Al: Bi_2_Te_3_ films, both damage threshold and transmittance demonstrate significant enhancement with increasing Al doping concentration. The third-order nonlinear optical absorption coefficient of 3.12% Al-doped Bi_2_Te_3_ thin films was 1.59 times higher than that of undoped Bi_2_Te_3_ thin films. These findings provide an effective way to develop nonlinear optical materials with excellent performance and promote the application potential of Bi_2_Te_3_ thin films in the field of optoelectronic devices.

## 2. Materials and Methods

Al-doped Bi_2_Te_3_ (Al: Bi_2_Te_3_) thin films were fabricated on both Si (100) and fused quartz substrates at room temperature by the magnetron co-sputtering system (Infovion EBAS, Bucheon-si, Republic of Korea). The background and working pressure are 6.00 × 10^−4^ Pa and 0.48 Pa, respectively, in the chamber. During the co-sputtering process, the Bi_2_Te_3_ target with a purity of 99.999% was used for radio frequency (RF) sputtering, while the Al target with a purity of 99.99% was employed for direct current (DC) sputtering. In order to regulate the content of Al in the films, the RF sputtering power was maintained at 50 W, and the DC sputtering power was set up to 0 W, 30 W, 40 W, and 50 W, respectively. Corresponding to the Al doping concentrations, the as-deposited film samples were designated as S0, S30, S40, and S50. The sputtering time for all samples was strictly controlled at 80 s.

The crystallization characteristics of undoped and Al-doped Bi_2_Te_3_ thin films were determined by the X-ray diffraction (XRD) (Bruker D2 PHASER, Karlsruhe, Germany) using Cu-Kα (λ = 1.54184 Å) radiation in the 2θ range from 10° to 60°, with a scanning step of 0.02°. The surface and cross-sectional morphology of the samples were examined using scanning electron microscopy (SEM) (ZEISS Gemini300, Jena, Germany). The magnification is ×10^5^, and the resolution is 0.7 nm @ 15 kV. The energy-dispersive X-ray spectroscopy (EDS) (OXFORD Xplore 30, High Wycombe, UK) was utilized to determine the elemental composition and distribution of the samples. X-ray photoelectron spectroscopy (XPS) (Thermo Scientific K-Alpha, Waltham, MA, USA) was performed to analyze the chemical states of Al-doped Bi_2_Te_3_ thin films. To investigate the effect of Al doping on the optical properties of Bi_2_Te_3_ thin films, the transmittance was measured by a double-beam UV/VIS/NIR spectrophotometer (Shimadzu UV-3600, Kyoto, Japan) in the wavelength range of 300–1500 nm. The optical constants of the sample films, including the refractive index and extinction coefficient in the visible range, were obtained by fitting ellipsometric spectroscopy data using the Film Wizard^®^ software version 6.10.1.

**Figure 1:**
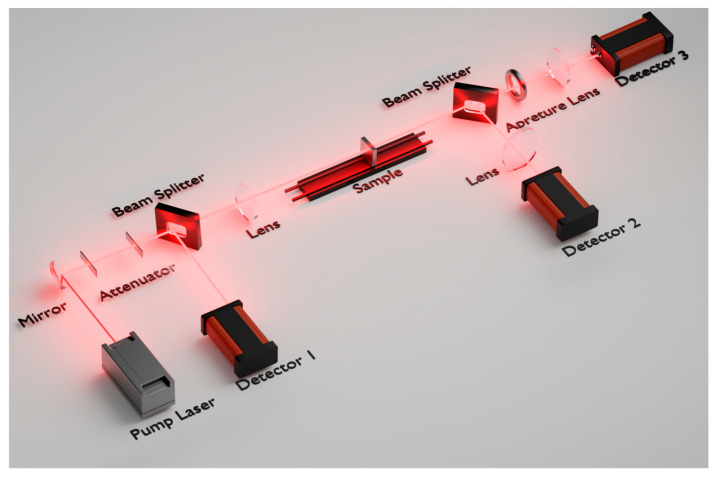
Schematic of Z-scan experimental setup.

The nonlinear optical absorption characteristics of all samples were investigated by a single-beam open-aperture Z-scan system. The experimental setup is shown in Figure 1. The open-aperture Z-scan was employed to characterize the nonlinear optical absorption effects of the thin films without the aperture in front of Detector 2. The Ti–sapphire regenerative amplifier system (Spectra Physics, Mountain View, CA, USA) with a pulse width of 100 fs, a repetition rate of 1 kHz, and a central wavelength of 800 nm served as the excitation source. The laser beam is focused through a convex lens with a focal length of 30 cm and is vertically incident onto the surface of the sample films. The beam radius *ω_0_* at the focal point was approximately 46.5 ± 1.2 μm. The laser power incident on the sample films was precisely controlled and continuously adjusted by two attenuators. The transmitted pulse laser power was directly recorded using an optical power meter (Newport 918D-SL-OD3R, Andover, MA, USA), providing real-time data for the nonlinear optical absorption analysis. The system was calibrated using a standard CS_2_ solution in a 1 mm thick quartz cuvette for closed-aperture Z-scan and ZnSe thin films for open-aperture Z-scan.

## 3. Results and Discussion

### 3.1. Structure, Morphology, and Chemical States Analysis

**Figure 2:**
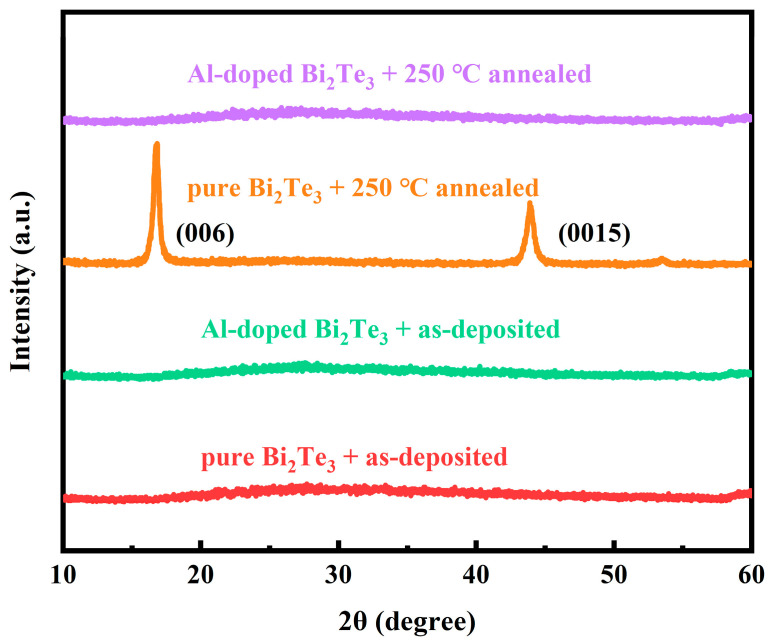
XRD patterns of Al: Bi_2_Te_3_ thin films.

The structural properties of the Al: Bi_2_Te_3_ thin films deposited on silicon substrates were analyzed by the XRD technique. As shown in Figure 2, the XRD patterns of the as-deposited undoped Bi_2_Te_3_ and Al-doped Bi_2_Te_3_ thin films exhibit no diffraction peaks, indicating an amorphous nature. After annealing at 250 °C in a vacuum (lower than 0.1 Pa) for 1 h, the undoped Bi_2_Te_3_ thin films display a rhombohedral crystal structure. The diffraction peaks corresponding to the (006) and (0015) planes align with JCPDS NO: 15-0863, indicating that the crystalline film preferentially grows along the c-axis and exhibits a high degree of crystallinity. In contrast, the Al-doped Bi_2_Te_3_ thin films annealed under the same conditions still show the same XRD pattern as the deposited film, with the absence of any crystalline diffraction peaks, indicating an amorphous state. This suggests that the Al dopant increases the defects and structural disorders in the thin films. The amorphous phase of the Al-doped Bi_2_Te_3_ thin films can remain stable up to an annealing temperature of 250 °C, which further increases its suitability for utilization in phase-change memory applications [11]. In previous studies, such as Mn-doped Bi_2_Te_3_ thin films [15], Ta-doped InTe thin films [18], and Al-doped Bi_2_Se_3_ nanoparticles [21], the decrease of crystallinity after doping was also observed, which confirmed the influence of dopant elements on the structure.

**Figure 3:**
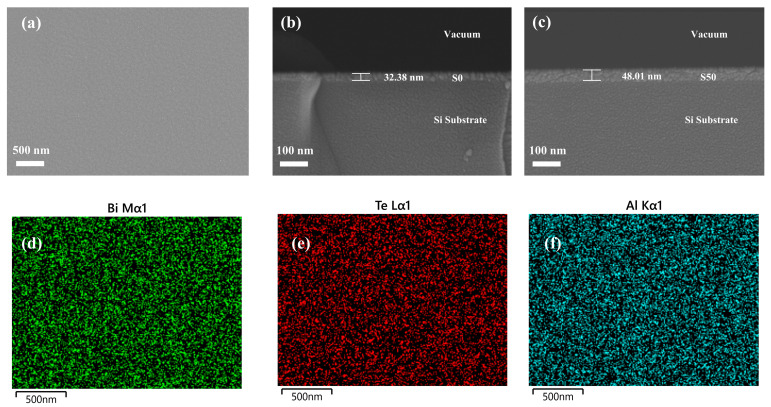
(**a**) The surface morphology of the S30 sample and cross-sectional SEM images of the (**b**) S0 and (**c**) S50 samples; element mapping images of the S30 sample for (**d**) Bi, (**e**) Te, and (**f**) Al.

As shown in Figure 3a, the surface morphology of the as-deposited Al-doped Bi_2_Te_3_ thin film (S30) is presented. The surface of the thin film is smooth and flat, without noticeable grains, exhibiting a typical amorphous thin film morphology. The thickness of the thin film was estimated from the SEM cross-sectional images. Figure 3b,c show the cross-sections of the undoped (S0) and Al-doped (S50) thin films, respectively. The upper vacuum environment, middle film layer, and Si substrate are distinguishable, with the film layer exhibiting uniform and continuous properties, indicating good film quality. The thin film thicknesses for the S0, S30, S40, and S50 samples with different Al doping concentrations were measured to be approximately 32.38 nm, 35.73 nm, 40.19 nm, and 48.01 nm, respectively. Furthermore, EDS was used to analyze the composition of Al: Bi_2_Te_3_ thin films. Table 1 lists the Al content in the sample films with different DC sputtering powers. By detecting the characteristic X-rays of specific elements, corresponding two-dimensional element distribution maps were generated to visually illustrate the distribution of Bi, Te, and Al of the films. As depicted in Figure 3d–f, Bi, Te, and Al elements are uniformly distributed throughout the Al-doped Bi_2_Te_3_ thin films.

**Figure 4:**
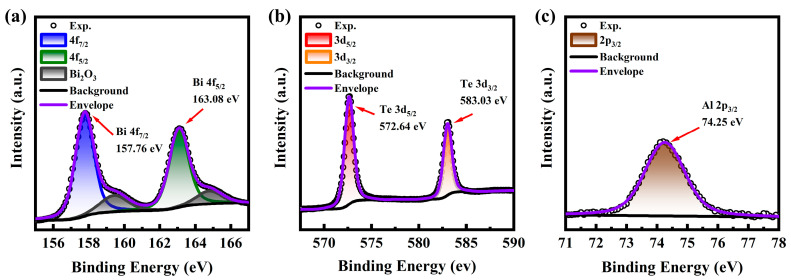
XPS spectrums of (**a**) Bi 4f (**b**) Te 3d and (**c**) Al 2p for the S30 sample.

XPS analysis was performed to further investigate the chemical states of the elements on the surface of the prepared Al: Bi_2_Te_3_ thin films. To correct for peak shifts caused by the charge effect, the C1s peak at a binding energy of 284.8 eV was used for spectrum calibration [22]. The core levels of the primary constituent elements (Bi, Te, Al) in the samples were extracted, including Bi 4f, Te 3d, and Al 2p. In order to precisely fit the peak shapes, the Gauss–Lorentz function was selected for peak fitting analysis, and the relevant results are shown in Figure 4a–c. In the high-resolution Bi spectrum (Figure 4a), two sets of doublets are observed: one at binding energies of 157.76 eV and 159.42 eV and the other at 163.08 eV and 164.78 eV. These doublets correspond to Bi 4f_7/2_ and Bi 4f_5/2_, respectively, with a spin-orbit splitting energy of 5.32 eV, which aligns with the characteristics of Bi^3+^ in the material [23]. The two dominant peaks at 157.76 eV and 163.08 eV originate from Bi in Bi_2_Te_3_, while the two weaker peaks at 159.42 eV and 164.78 eV are associated with the Bi–O bond, indicating the presence of Bi_2_O_3_ due to surface oxidation [22]. In the high-resolution Te 3d spectrum (Figure 4b), two prominent peaks are observed at binding energies of 572.64 eV and 583.08 eV, corresponding to the Te 3d_5/2_ and 3d_3/2_ spin states, respectively. These peaks suggest that Te exists in the form of Te^2-^ in the sample films. Compared with the undoped Bi_2_Te_3_ film, the binding energy of Te in the doped samples exhibits a slight shift to higher values (0.18 eV), indicating that the chemical environment of Te ions is affected by Al doping [24]. Figure 4c shows the Al 2p spectrum, where the Al 2p_3/2_ peak is observed at the binding energy of 74.25 eV. Notably, the metallic Al peak, which is typically centered at 72.4 eV, is not found [25], confirming that Al doping primarily occurs in the Al^3+^ state [26].

### 3.2. Linear Optical Analysis

The linear optical properties of thin films are crucial in determining their functionality in optoelectronic devices. To investigate the impact of Al doping on the linear optical properties of Al: Bi_2_Te_3_ thin films, a systematic study of optical constants was conducted using a spectroscopic ellipsometer. Based on the structure of the thin films, a three-layer model (air-film-substrate) was established, and the Drude–Lorentz oscillator was employed to describe the dielectric response of the Al-doped Bi_2_Te_3_ thin films. The refractive index (*n*) and extinction coefficient (*k*) were obtained through spectral fitting. The Drude–Lorentz model is given by the following Equation (1) [27].

(1) $$\varepsilon_{\mathrm{DL}}=\varepsilon_{\infty }-\frac{\omega_{P}^{2}}{\omega^{2}+i\omega \gamma }+\sum_{k}\frac{C_{k}}{\omega_{k}^{2}-\omega \left(\omega +i\gamma_{k}\right)}$$

**Figure 5:**
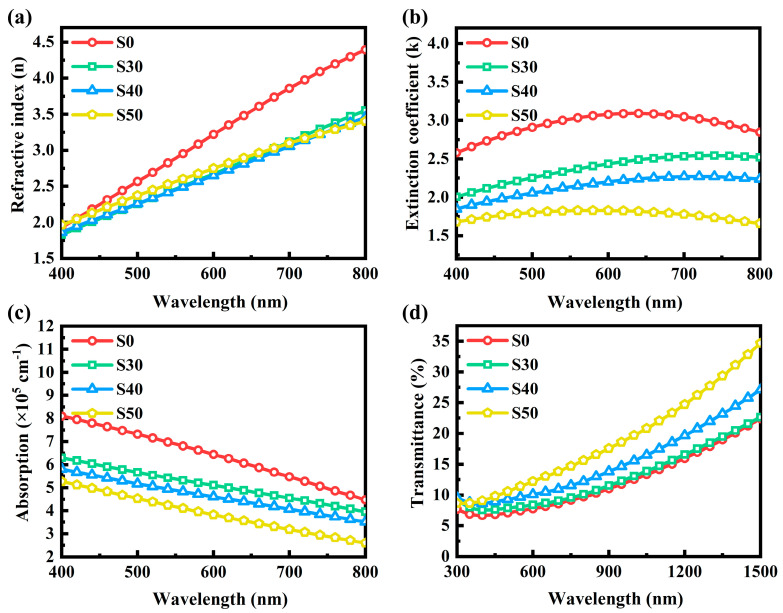
The (**a**) *n*, (**b**) *k* curves, (**c**) absorption spectra, and (**d**) transmission spectra of Al: Bi_2_Te_3_ thin films.

The *ε*_∞_ of the first term represents the dielectric response due to non-atomic polarization at high optical frequencies. The second term denotes the Drude model that studies the absorption of free carriers in a metal or semiconductor. The third term is given by the Lorentz model, which is typically associated with interband transitions of bound electrons. To ensure the accuracy of the experimental results, multiple measurements were carried out at incident angles of 65°, 70°, and 75°, from which the thickness, *n*, and *k* of the Al: Bi_2_Te_3_ thin films were determined. The fitted thicknesses of the S0, S30, S40, and S50 samples were 31.91 nm, 36.25 nm, 39.94 nm, and 48.45 nm, respectively, which are consistent with the cross-sectional thicknesses measured by SEM, with an error margin within ±1 nm. The results of *n* and *k* in the visible range are shown in Figure 5a,b. Al doping leads to a decrease in both *n* and *k* for the Bi_2_Te_3_ thin films, and the higher the Al concentration, the lower the *k* value. This phenomenon will be attributed to the smaller radius of Al^3+^ ions, which causes the distortion of the film lattice upon doping, leading to the generation of local stress or defects [28]. These factors can affect the film’s density and photon absorption, thereby changing its optical properties.

The linear absorption coefficient (*α*) of the Al: Bi_2_Te_3_ thin films can be calculated from the *k* value using the following Equation (2).

(2) α =4πk/λ

The *α* of the Al: Bi_2_Te_3_ thin films, shown in Figure 5c, gradually decreases with the increase in Al doping concentration. To verify the reliability of the data, a further test was conducted on the transmittance of the Al: Bi_2_Te_3_ thin films using a UV/VIS/NIR spectrophotometer, as depicted in Figure 5d. The transmittance curves for all samples exhibit similar shapes, with relatively low transmittance in the visible light band, which increases as the wavelength extends into the near-infrared region. As the Al doping concentration rises, the transmittance follows an inverse trend to the linear absorption coefficient. A similar phenomenon has been observed in Mn [15] and Cs [29] doped Bi_2_Te_3_ films. The higher transmittance effectively reduces energy loss, thus enhancing the optical efficiency and overall performance of devices.

Since Bi_2_Te_3_ thin films are direct bandgap semiconductor materials [30], the optical bandgap (*E_g_*) of the thin films can be calculated using the Tauc relation (3).

(3) $$\;\alpha h\nu =C{(h\nu -E_{g})}^{\frac{1}{2}}$$

**Figure 6:**
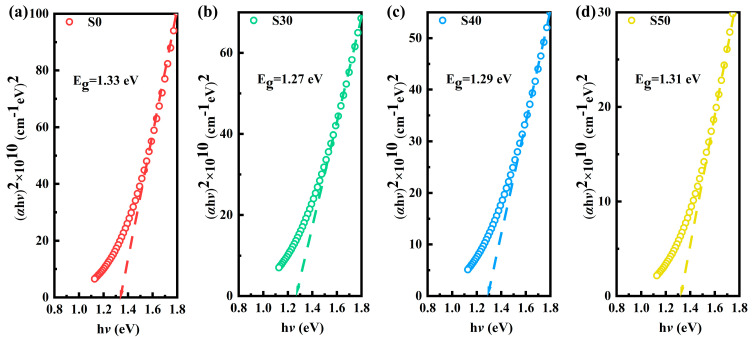
The optical bandgap of Al: Bi_2_Te_3_ thin films: (**a**) S0, (**b**) S30, (**c**) S40, (**d**) S50.

Here, *α* is the linear absorption coefficient, h*ν* refers to the incident photon energy, and C is a constant related to the effective mass. According to the Tauc method, the *E_g_* of the thin films can be determined by extrapolating the linear part of the (*α*h*ν*)^2^ vs. h*ν* curve to h*ν* = 0, as shown in Figure 6a–d.

The *E_g_* of undoped Bi_2_Te_3_ thin films is 1.33 eV, and it decreases significantly after Al doping. The observed decrease in optical bandgap following the introduction of Al dopants can be attributed to the increase of lattice defects and structural disorder within the thin films. These local defects create additional energy levels in the material, expanding the edges of the conduction band and the valence band, and form the Urbach band tail. This, in turn, affects the excitation energy for electron transitions from the valence band to the conduction band, resulting in a reduction in the *E_g_* [31]. The width of the Urbach tail (*E_u_*) can be expressed as the following formula [32].

(4) $$\ln\alpha =\frac{h\nu }{E_{u}}+C$$

**Figure 7:**
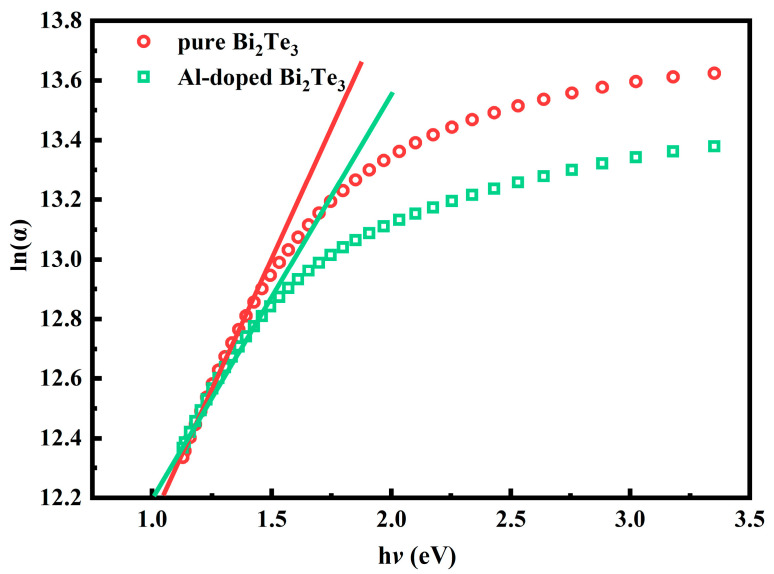
The plot of ln(*α*) as a function of the photon energy (h*ν*) for Al: Bi_2_Te_3_ thin films.

C is a constant, and *E_u_* can be interpreted as the width of the tails for localized states in the bandgap region, which can be estimated by plotting the ln(*α*) versus h*ν* curve, as shown in Figure 7. The calculated *E_u_* values for the undoped and Al-doped Bi_2_Te_3_ thin films are 0.58 eV and 0.71 eV, respectively. The increased structural disorder in the Al-doped films led to an increase in band tails and a decrease in the optical bandgap.

Moreover, with the increase in Al doping concentration, the *E_g_* of the Al: Bi_2_Te_3_ thin films further increases. The *E_g_* of the S30, S40, and S50 samples are 1.27 eV, 1.29 eV, and 1.31 eV, respectively. This phenomenon can be explained by the Burstein–Moss effect. As a donor dopant, Al leads to an increase in carrier concentration, which causes the Fermi level to shift upward into the conduction band. Consequently, the lower-energy electronic states in the conduction band become occupied, thereby increasing the energy required for electron transitions from the valence band to the conduction band. This effect causes a blue shift in the optical bandgap [33]. The result indicates that there is a competitive relationship between the Urbach tail and the Burstein–Moss effect during the doping process.

It was found that Al doping not only modifies the optical properties of Al: Bi_2_Te_3_ thin films but also affects their bandgap structure. The optical parameters such as refractive index, extinction coefficient, transmittance, and optical bandgap of the Al: Bi_2_Te_3_ thin film can be systematically regulated by varying the Al doping concentration. This finding provides a new idea for optimizing the optical properties of Bi_2_Te_3_ thin films and provides the necessary experimental data for further exploring its nonlinear optical properties.

### 3.3. Nonlinear Optical Analysis

**Figure 8:**
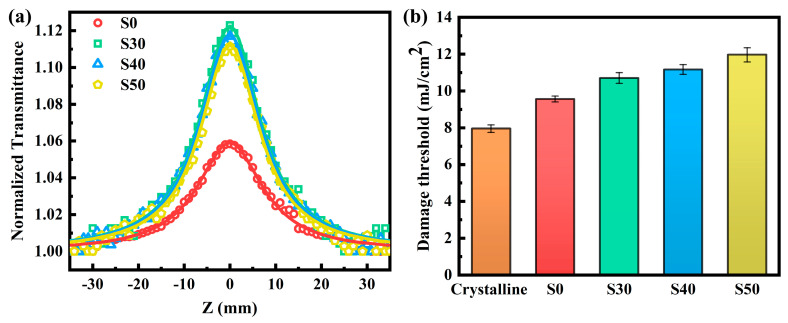
(**a**) Open-aperture z-scan curves and (**b**) damage thresholds of the Al: Bi_2_Te_3_ thin films.

In the field of nonlinear optics, the absorption of samples can be defined as *α_tot_* (*I*) = *α* + *βI*, where *α_tot_*, *α*, *β*, and *I* represent the total absorption coefficient, linear absorption coefficient, nonlinear optical absorption coefficient, and incident light intensity, respectively. To investigate the ultrafast nonlinear optical absorption characteristics of Al: Bi_2_Te_3_ thin films on fused quartz substrates, the single beam open-aperture Z-scan technique proposed by Sheik-Bahae [34] was employed, utilizing 800 nm femtosecond laser pulses. The nonlinear optical transmittance of a blank fused quartz substrate was measured in advance, showing no nonlinear optical absorption signal even if the peak input intensity reached about 2000 GW/cm^2^. To avoid interference from nonlinear scattering on the film surface and nonlinear optical responses of the substrate, all samples were tested at a relatively low incident power density of 33 GW/cm^2^. Under this experimental condition, *β* only represents the third-order nonlinear optical absorption coefficient, as the higher-order nonlinear optical absorption response can be neglected at low excitation laser intensities. The open-aperture Z-scan normalized transmittance curves of Al: Bi_2_Te_3_ thin films with different Al doping concentrations are shown in Figure 8a. All samples displayed a sharp and centrosymmetric peak at the focal point, reflecting the saturable absorption behavior. Since the photon energy of the laser source (E = 1.55 eV) is higher than the optical bandgap of the Al: Bi_2_Te_3_ thin films (1.27~1.33 eV), high-energy photons can directly excite electrons from the valence band to the conduction band. According to the Pauli exclusion principle, lower-energy states in the conduction band are filled first. With increasing pulse intensity, more electrons are excited to the conduction band, resulting in the rapid depletion of electrons in the valence band and the occurrence of the ground state bleaching effect. Furthermore, as the lower-energy states at the bottom of the conduction band become increasingly occupied, the effective absorption of the film diminishes when the photon energy is insufficient to excite electrons to higher-energy states, leading to an increase in transmittance. At the same excitation laser intensity, the Al-doped Bi_2_Te_3_ thin films exhibited enhanced nonlinear transmittance, suggesting a higher modulation depth. Particularly in mode-locked lasers, a high modulation depth can significantly suppress pulse wave-breaking effects and provide strong pulse shaping, leading to shorter pulse durations and more stable self-starting behavior [35].

The value of *β* can be obtained by fitting the data collected from the open-aperture Z-scan system using Equation (5) [36].

(5) $$T_{\mathrm{OA}}\left(z\right)=\sum_{m=0}^{\infty }\frac{{\left[-\beta I_{0}L_{\mathrm{eff}}/(1+z^{2}/z_{0}^{2})\right]}^{m}}{{\left(m+1\right)}^{3/2}}$$

Here, *T_OA_* represents the normalized transmittance, *β* denotes the third-order nonlinear optical absorption coefficient, *I*_0_ is the laser intensity at the focal point, and z is the distance from the sample to the focus. *Z*_0_ refers to the Rayleigh length, which can be calculated using the wavelength (λ) and the waist radius (*ω*_0_) of the Gaussian beam.

(6) $$Z_{0}=\frac{\pi \omega_{0}^{2}}{\lambda }$$

*L_eff_* is defined as the effective thickness of the films, as given by Equation (7), where L represents the physical thickness of the thin films.

(7) $$L_{\mathrm{eff}}=\frac{\left(1-e^{-\alpha_{0}L}\right)}{\alpha_{0}}$$

The value of *β* obtained by fitting is negative, indicating that the samples exhibit a lower total absorption coefficient and higher transmittance under high excitation laser intensity. It is worth noting that the magnitude of *β* for Al-doped Bi_2_Te_3_ thin films is larger than for undoped Bi_2_Te_3_ thin films and gradually decreases with increasing Al doping concentration. This trend correlates well with the variation in optical bandgap (Figure 6), indicating that a smaller optical bandgap facilitates saturation. The same phenomenon was also found in amorphous InSe thin films [37], and it has been demonstrated that nonlinear effects are more pronounced in materials with smaller optical bandgaps [38]. In addition, Al doping increases the defect concentration, and more carriers get trapped in defect states [39]. The corresponding carrier recombination lifetime is prolonged, enhancing the ground state bleaching effect and further improving saturable absorption performance [40].

Multiple tests were conducted on the Al: Bi_2_Te_3_ thin films, and the results remained within a reliable error range, confirming the reliability and repeatability of the experimental data. The imaginary part of third-order nonlinear optical susceptibility (Im*χ*^(3)^) can be further determined through the following relation (8).

(8) Imχ3=10−7cλn296π2β

To eliminate the discrepancy caused by linear optical absorption, the saturable absorption performance of Al: Bi_2_Te_3_ thin films can be evaluated using the figure of merit (FOM).

(9) FOM =Imχ(3)/α

Table 2 summarizes all nonlinear optical absorption parameters for Al: Bi_2_Te_3_ thin films.

The damage threshold refers to the maximum laser energy density that the thin film can endure without sustaining permanent damage under high-power laser irradiation. The damage threshold of thin film directly impacts the reliability and safety of the whole system. Figure 8b displays the damage threshold of crystalline Bi_2_Te_3_ thin films, amorphous Bi_2_Te_3_ thin films (S0), and amorphous Al-doped Bi_2_Te_3_ thin films with varying doping concentrations (S30, S40, and S50) from left to right. It is observed that the amorphous thin films exhibit higher damage thresholds. Specifically, as the Al doping concentration increases, the damage threshold rises from 9.56 ± 0.16 mJ/cm^2^ to 11.97 ± 0.39 mJ/cm^2^. This enhancement is primarily attributed to the reduction in the absorption coefficient of the thin films, which mitigates localized thermal accumulation under laser irradiation.

It has been proved that different forms of Bi_2_Te_3_ materials fabricated by various methods exhibit significant saturable absorption characteristics, such as bulk-structured Bi_2_Te_3_ [9], Bi_2_Te_3_ nanoparticles [41], and Te-Bi_2_Te_3_ alloy thin film [42]. In recent years, with the growing demand for ultrafast optical devices, enhancing their nonlinear optical absorption response has become a critical challenge to promote the development of this field. As summarized in Table 3, Al-doped Bi_2_Te_3_ thin films exhibit comparable or superior saturable absorption performance compared with previously reported advanced materials.

Al-doped Bi_2_Te_3_ thin films exhibit a strong third-order nonlinear optical absorption response under excitation by an 800 nm femtosecond pulsed laser, making them promising candidates for applications in all-optical angular momentum devices [50]. Both high modulation depth and damage threshold are key parameters for generating laser pulses with large energy. The Al doping enhances the saturable absorption characteristics of amorphous Bi_2_Te_3_ thin films while simultaneously improving their damage thresholds and optical transmittance. Furthermore, systematic adjustment of Al doping concentrations enables precise regulation of linear optical properties. These findings demonstrate the substantial application potential of Al: Bi_2_Te_3_ thin films in passive mode-locked lasers, Q-switched lasers, and all-optical diodes.

## 4. Conclusions

In summary, the amorphous Al: Bi_2_Te_3_ thin films with varying Al doping concentrations were deposited on both fused quartz and Si substrates using a magnetron co-sputtering technique. The structure, morphology, linear optical and nonlinear optical absorption properties of Al: Bi_2_Te_3_ thin films were investigated in detail. XRD measurements confirmed the amorphous nature of Al: Bi_2_Te_3_ thin films. SEM images illustrated that the film thickness increased with higher Al doping concentrations, and the surface of the Al-doped Bi_2_Te_3_ films was smooth and flat without any visible grains, displaying a typical amorphous morphology. Chemical analysis revealed that the Al dopant was present in the Al: Bi_2_Te_3_ thin films as Al^3+^. Regarding the linear optical properties, the refractive index and extinction coefficient of the films decreased with increasing Al doping concentrations, while the measured transmittance was improved. Due to the competition between the Urbach tail and the Burstein–Moss effect, the optical bandgap of the Al-doped Bi_2_Te_3_ thin films was reduced compared with the undoped Bi_2_Te_3_ thin films. However, it increased with higher Al doping concentrations. The nonlinear optical absorption response of the Al: Bi_2_Te_3_ thin films was investigated using an open-aperture Z-scan system, and all samples exhibited saturable absorption characteristics. Al doping significantly improved the nonlinear optical absorption coefficient and modulation depth for the Bi_2_Te_3_ thin films. This enhancement is attributed to the increased defect state density induced by Al doping, which is closely related to the optical bandgap of the films. Furthermore, the damage threshold increased from 9.56 ± 0.16 mJ/cm^2^ to 11.97 ± 0.39 mJ/cm^2^ with Al doping. The results of this study demonstrate that the optical properties of Al: Bi_2_Te_3_ thin films, such as refractive index, extinction coefficient, and optical bandgap, can be flexibly modulated by adjusting the Al doping concentration. Compared with other methods, such as annealing treatment and heterostructure fabrication, Al doping not only effectively enhances the third-order nonlinear optical absorption coefficient of Bi_2_Te_3_ thin films but also simultaneously improves their optical transmittance and damage thresholds. These findings indicate that Al-doped Bi_2_Te_3_ thin film is an excellent saturable absorber material and has potential applications in all-optical switching, mode-locked lasers, and other fields.

## Figures and Tables

**Table 1:** The composition and thickness of the Al: Bi_2_Te_3_ thin films.

Samples	Al Target Power (W)	Al (wt.%)	Thickness (nm)
S0	0	0	32.38
S30	30	3.12	35.73
S40	40	5.28	40.19
S50	50	8.93	48.01

**Table 2:** The nonlinear optical absorption parameters for Al: Bi_2_Te_3_ thin films by Z-scan.

Samples	*L_eff_* (nm)	*β* (cm/GW)	Im*χ*^(3)^ (×10^−9^ esu)	FOM (10^−14^ esu × cm)	Damage Threshold (mJ/cm^2^)
S0	16.99 ± 0.09	−2940.94 ± 18.23	−14.39 ± 0.09	3.22 ± 0.02	9.56 ± 0.16
S30	19.25 ± 0.13	−4664.52 ± 34.37	−14.91 ± 0.11	3.78 ± 0.03	10.7 ± 0.29
S40	24.18 ± 0.16	−4031.62 ± 32.06	−12.19 ± 0.1	3.47 ± 0.03	11.17 ± 0.26
S50	27.53 ± 0.22	−2958.56 ± 36.48	−8.67 ± 0.11	3.33 ± 0.04	11.97 ± 0.39

**Table 3:** Comparison of the nonlinear optical parameters for different forms of Bi_2_Te_3_ materials and other advanced materials.

Materials	λ (nm)	*β* (cm/GW)	Synthesis Method	Reference
Graphene	790	−90~−20	oxidation-reduction method	[43]
Graphene-PVA	1030	−(0.66 ± 0.3)	solution cast method	[44]
MoS_2_	800	−(2.42 ± 0.8) × 10^−2^	liquid-phase exfoliation	[45]
MoTe_2_	800	−(3.7 ± 1.2) × 10^−3^	liquid-phase exfoliation	[45]
Au-decorated MoS_2_/PEDOT: PSS	532	−(3.5 ± 0.23) × 10^3^	ultrasonic technique	[46]
Bi_2_Se_3_ nanosheets	800	−1.67 × 10^3^	solution-based methods	[47]
Bi_2_Te_3_ colloidal suspensions	1064	−70	ultrasonic bath method	[41]
Bi_2_Te_3_ nanoplates	800	−5.7 × 10^−4^	selective metal deposition	[48]
Bi_2_Te_3_-FeTe_2_ nanoplates	800	−7.53 × 10^−4^	selective metal deposition	[48]
Bi_2_Te_3_ thin film	532	−80	solvent-heat method	[49]
Al: Bi_2_Te_3_ thin film	800	−(4664.52 ± 34.37)~−(2940.94 ± 18.23)	magnetron co-sputtering	This work

## Data Availability

The original contributions presented in the study are included in the article, further inquiries can be directed to the corresponding author.
